# Direct, diffuse and total solar radiation data set in La Guajira, Magdalena and Cesar departments -Colombia

**DOI:** 10.1016/j.dib.2020.106397

**Published:** 2020-10-15

**Authors:** Marley Vanegas Chamorro, Edwin Espinel Blanco, Jhan Piero Rojas

**Affiliations:** a Facultad de Ingeniería, Grupo de investigación en gestión eficiente de la energía – kaí. Universidad del Atlántico, Carrera 30 Número 8-49, Área Metropolitana de Barranquilla, 080007 Puerto Colombia, Colombia; b Facultad de Ingeniería, Universidad Francisco de Paula Santander, Vía Acolsure. Sede el Algodonal Ocaña, Ocaña-Norte de Santander 546552, Colombia; c Facultad de Ingeniería, Universidad Francisco de Paula Santander, Avenida Gran Colombia No. 12E-96, Cúcuta 540003, Colombia

**Keywords:** Solar radiation, Transmittance, Bird and Hulstrom model, Solarimetric information

## Abstract

This article aims to show direct, diffuse, and total solar radiation in the departments of La Guajira, Magdalena, and Cesar, located on the Caribbean coast of Colombia. In addition, data on climatic variables such as temperature, pressure, and relative humidity measured through different sensors located in these meteorological stations are presented. The data obtained by these stations correspond to measurements from 1993 to 2013 allowed the estimation of the parameters of the total, direct and diffuse solar radiation for each department, by mean of the Bird and Hulstrom model and parameterizations of the Mächler and Iqbal model. In addition, five climatological scenarios that could occur using these data were calculated.

## Specifications Table

**Table:** 

Subject area	Renewable energy
More specific subject area	Solar field, solar energy
Type of data	Raw, Graphs, figures, tables
How data was acquired	Pressure sensor Lambrecht Ref. 8121, temperature sensor Siap + Micros Ref. T001-TTEP-N and relative humidity Siap + Micros Ref. T003-TEH-V.
Data format	Raw data and analyzed
Parameters for data collection	The data mentioned in this article was administrated by the stations managed by the Hydrology, Meteorology, and Environmental Studies Institute (IDEAM).
Description of data collection	The data was collected through the sensors in the weather stations. This instrumentation acquires data every day and is recorded in real-time in the data acquisition system.
Data source location	Caribbean region in Colombia, Departments of La Guajira, Magdalena and Cesar.
Data accessibility	Repository name: Mendeley data: https://data.mendeley.com/datasets/hytc559th5/1 Data identification number: 10.17632/hytc559th5.1
Related research article	M. Vanegas, O. Churio, G. Valencia, E. Villicaña, and A. Ospino, “Calculation of total, direct and difusse radiation, through the atmospheric transmissivity in the departments of Cesar, La Guajira and Magdalena (Colombia),” Revista Espacios, vol. 38, no. 7, 2017 [1].

## Value of the Data

• The data provided in this article can be used as a starting point for further research on the behavior of solar radiation in the country, specifically in the Caribbean region of Colombia.
• The raw data presented can be used for the validation of new irradiation calculation models, either empirical or through neural networks.
• These data can be used to make a comparative analysis of solar potential with respect to different regions of the world.

## 1 Data Description

**Fig. 1:**
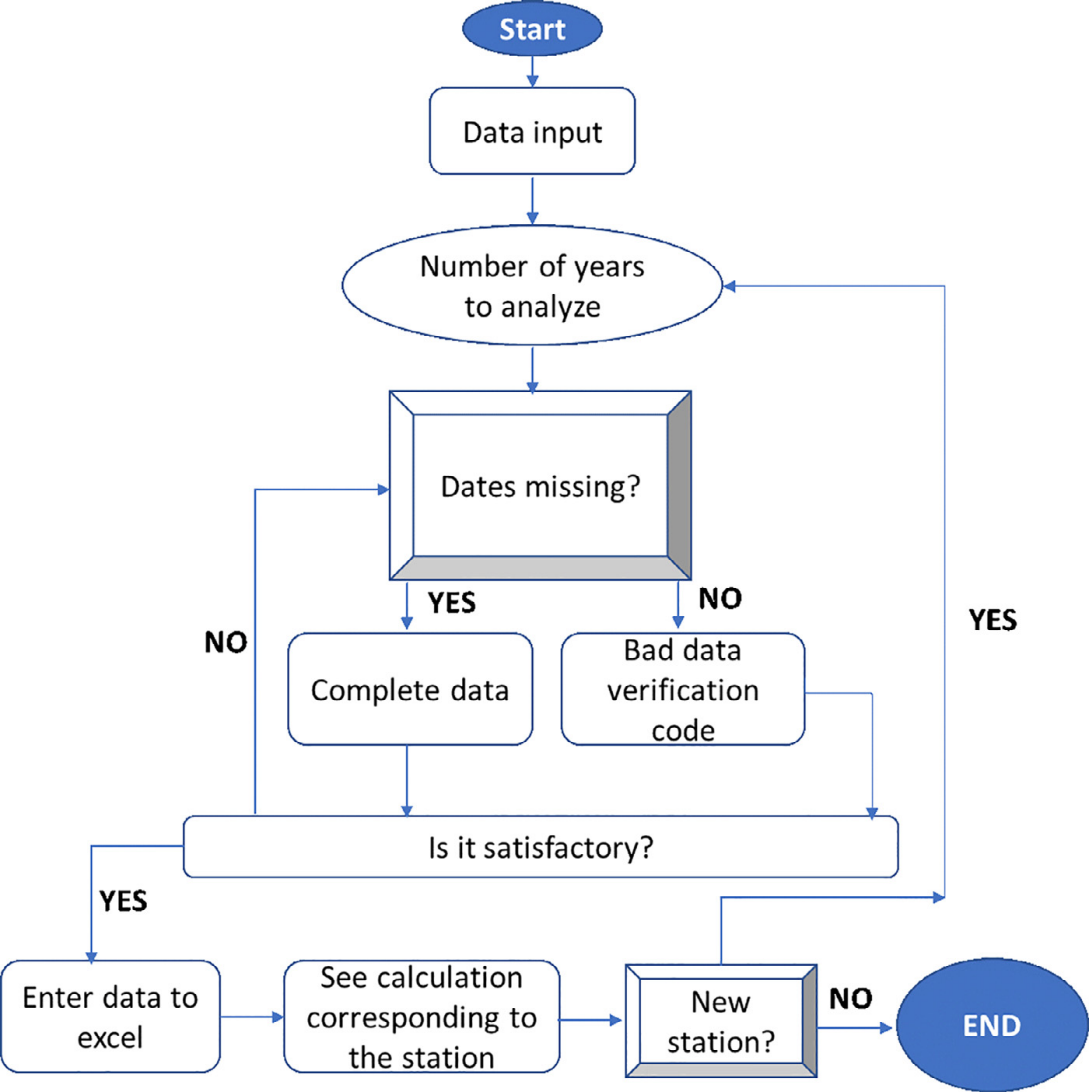
Flowchart for the treatment of meteorological data.

The data shown in this article are climatic measurements obtained from the stations located in the departments of La Guajira, Magdalena, and Cesar (Colombia). They were obtained from daily averages for each station, from which the direct (IDH), diffuse (IdH) and total (ITH) solar radiation data for different atmospheres were calculated using the Bird and Hulstrom model [2]. This model makes it possible to quantify the different atmospheric transmittances (τ) that are required for the calculation of radiation using the Angström turbidity coefficient (β). This coefficient allows analyzing radiation in different types of atmosphere: (β = 0.0) extremely clean, (β = 0.1) clear, (β = 0.2) medium, (β = 0.3) cloudy, and (β = 0.4) very cloudy. To complete the missing data, it was necessary to apply an interpolation of the available measures as described in Fig. 1. Therefore, daily averages of temperature and humidity were generated for each location in the departments under study.

**Fig. 2:**
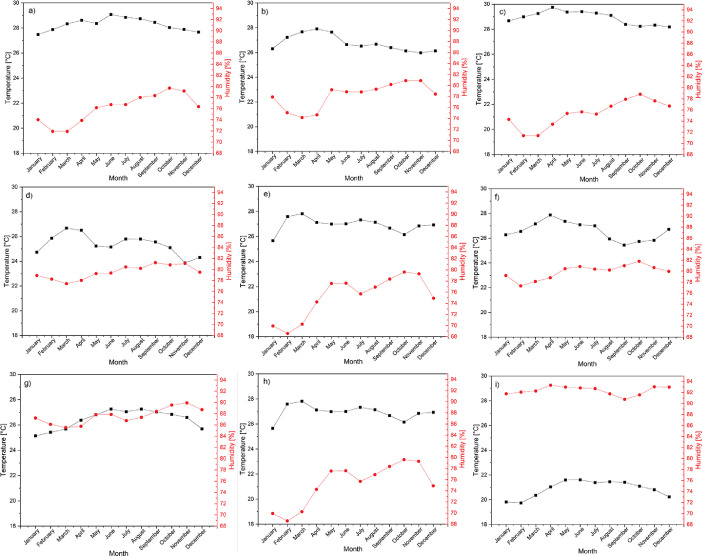
Temperature and humidity for weather stations in Magdalena from 1993 to 2013, (a) Apto Simón Bolívar, (b) Prado Sevilla, (c) La Ye, (d) Padelma, (e) Media Luna, (f) Los Álamos, (g) Tayrona, (h) EL seis, and (i) Alto de Mira.

**Fig. 3:**
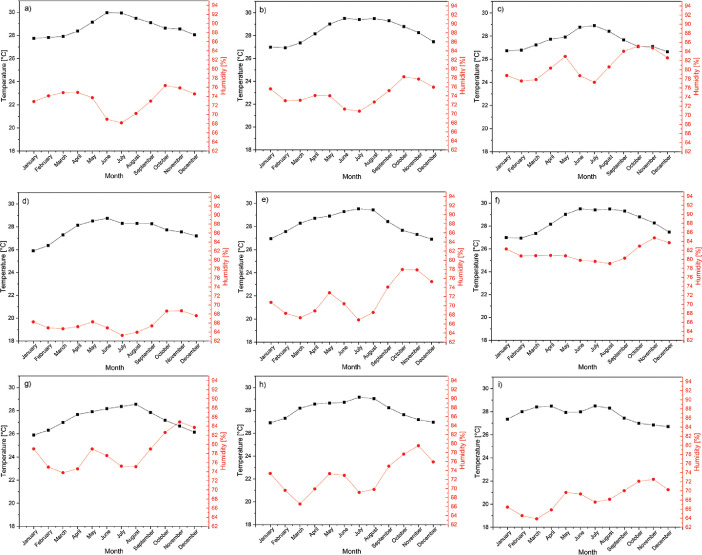
Temperature and humidity for weather stations in La Guajira from 1993 to 2013, (a) Manaure, (b) Pto. Bolivar, (c) Matitas, (d) Rancho Grande, (e) La Mina, (f) Nazareth, (g) Carraipía, (h) Camp. Intercor, and (i) Urumita.

**Fig. 4:**
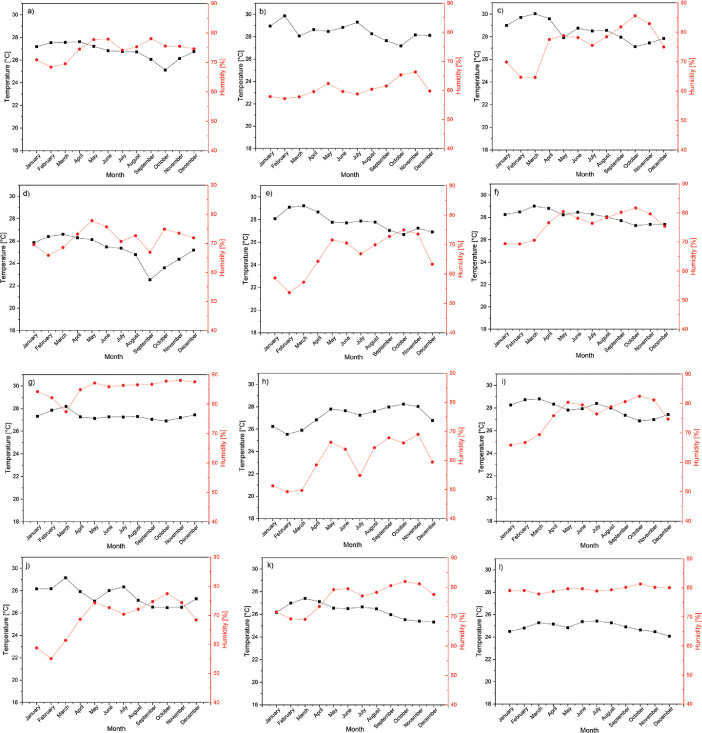
Temperature and humidity for weather stations in Cesar from 1993 to2013, (a) Chiriguaná, (b) Guaymaral, (c) Hda. La Guaira, (d) Col. Agro. Pailitas, (e) Villa Rosa, (f) Centenario Hda, (g) La Llana, (h) Apto Alfonso López, (i) Somba, (j) Motilonia Codazzi, (k) El Rincón and (l) San José Oriente.

The average climatic variables for each department are presented in Table 1 (Magdalena), Table 2 (La Guajira), and Table 3 (Cesar). Besides, the monthly trends in temperature and humidity in these places are presented in Fig. 2 (Magdalena), Fig. 3 (La Guajira), and Fig. 4 (Cesar).

**Table 1:** Climate variables for the weather stations in the Magdalena department.

N°	Name	Elevation (msnm)	Pressure (Pa)	HR* (%)	T⁎⁎ ( °C)
1	Apto Simón Bolívar	4	101278.5	76.1	28.3
2	Prado Sevilla	18	101115.8	78.2	26.8
3	La ye	20	101092.6	75.4	28.9
4	Padelma	20	101092.6	79.5	25.4
5	Media Luna	20	101092.6	79.9	26.6
6	Los Álamos	25	101034.6	79.9	28.2
7	Tayrona	30	100976.6	87.6	26.4
8	El Seis	50	100745.1	75.2	26.9
9	Alto de Mira	1080	89509.9	92.3	209

⁎ : relative humidity.

⁎⁎ : temperature.

**Table 2:** Climate variables for the weather stations in the La Guajira department.

N°	Name	Elevation (msnm)	Pressure (Pa)	HR* (%)	T⁎⁎ ( °C)
1	Manaure	1	101313.4	73.1	28.7
2	Pto. Bolívar	10	101208.7	74.2	28.4
3	Matitas	20	101092.6	80.9	27.6
4	Rancho Grande	50	100745.1	65.8	27.7
5	La Mina	80	100398.7	71.6	28.3
6	Nazareth	85	100341.1	81.3	27.2
7	Carraipía	118	99961.7	78.3	27.3
8	Camp. Intercor	122	99915.8	72.7	28.0
9	Urumita	255	98401.8	68.4	27.7

⁎ : relative humidity.

⁎⁎ : temperature.

**Table 3:** Climate variables for the weather stations in the Cesar department.

N°	Name	Elevation (msnm)	Pressure (Pa)	HR* (%)	T⁎⁎ ( °C)
1	Chiriguaná	40	100860.8	74.3	26.8
2	Guaymaral	50	100745.1	60.5	28.5
3	Hda. La Guaira	50	100745.1	76.1	28.5
4	Col. Agro. Pailitas	50	100745.1	71.7	25.2
5	Villa Rosa	70	100514.0	66.4	27.8
6	Centenario Hda	100	100168.4	76.4	28.1
7	La Llana	120	99938.7	85.4	27.3
8	Apto Alfonso López	138	99732.4	60.0	19.4
9	Socomba	170	99366.7	76.0	27.9
10	Motilonia Codazzi	180	99252.7	69.0	27.6
11	El Rincón	350	97334.5	76.5	26.3
12	San José de Oriente	850	91904.8	79.5	24.9

⁎ : relative humidity.

⁎⁎ : temperature.

**Fig. 5:**
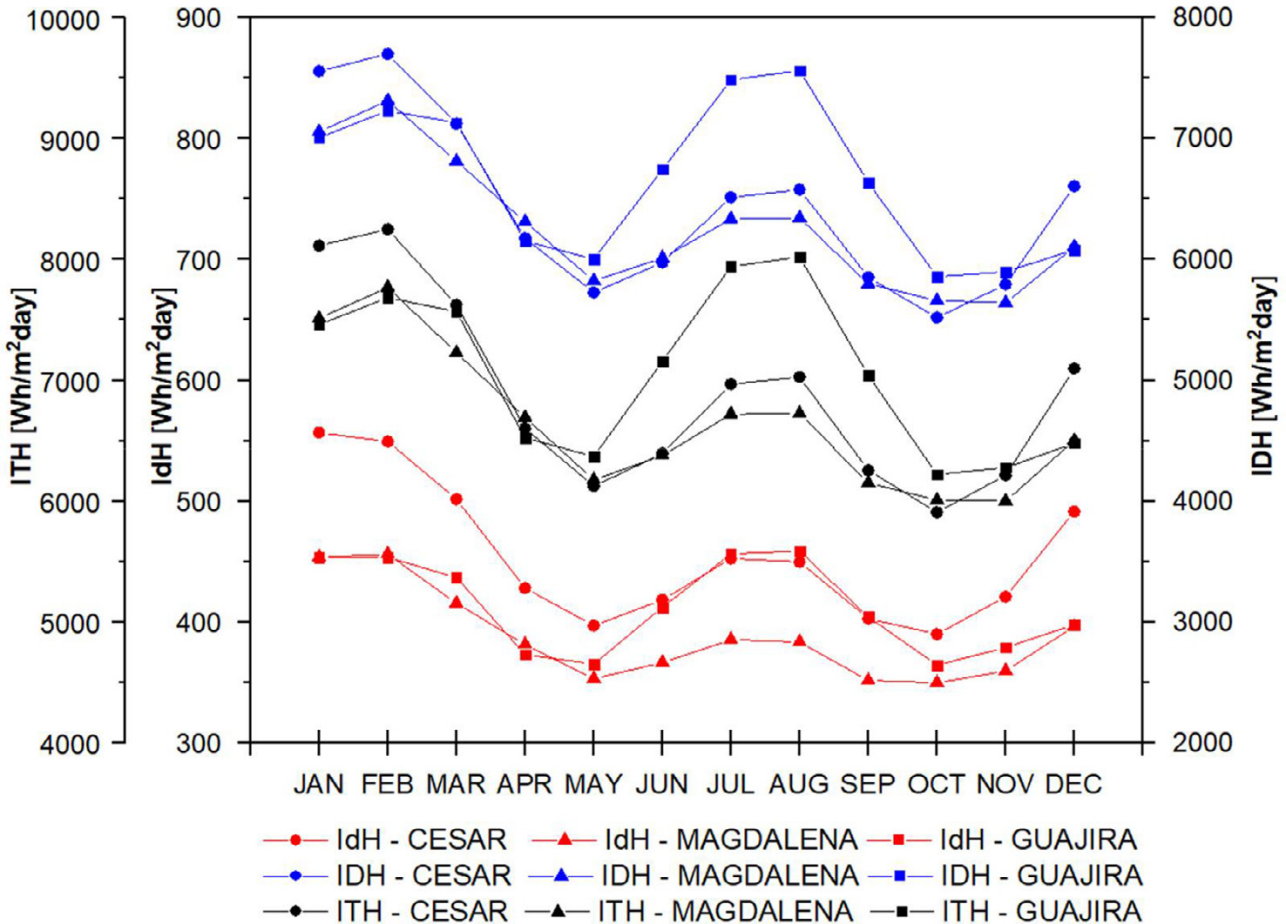
Analysis of direct, diffuse and total radiation at La Guajira, Magdalena and Cesar weather stations from 1993 to 2013 for a *τ*(0.0).

**Fig. 6:**
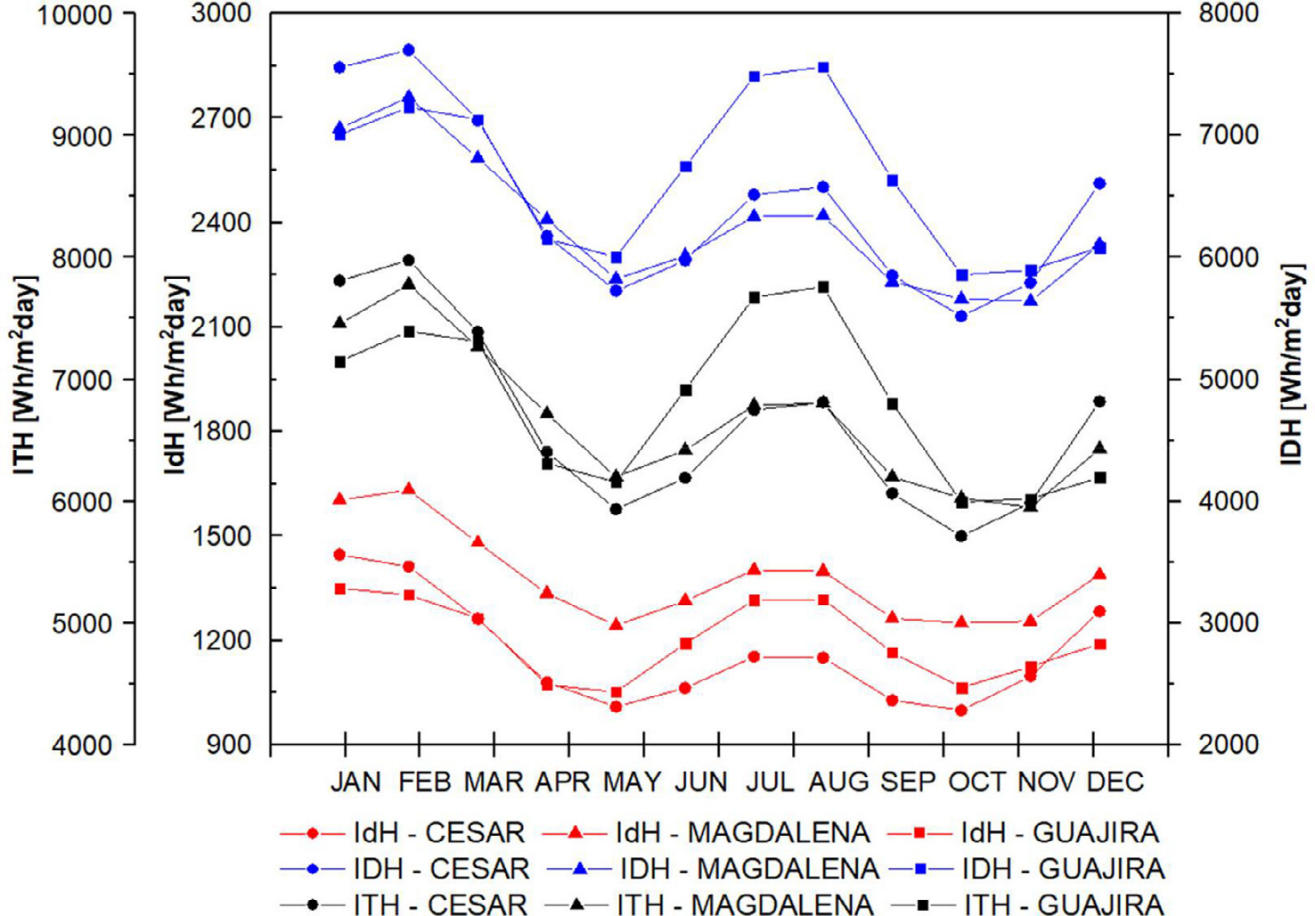
Analysis of direct, diffuse and total radiation at La Guajira, Magdalena and Cesar weather stations from 1993 to 2013 for a *τ*(0.1).

**Fig. 7:**
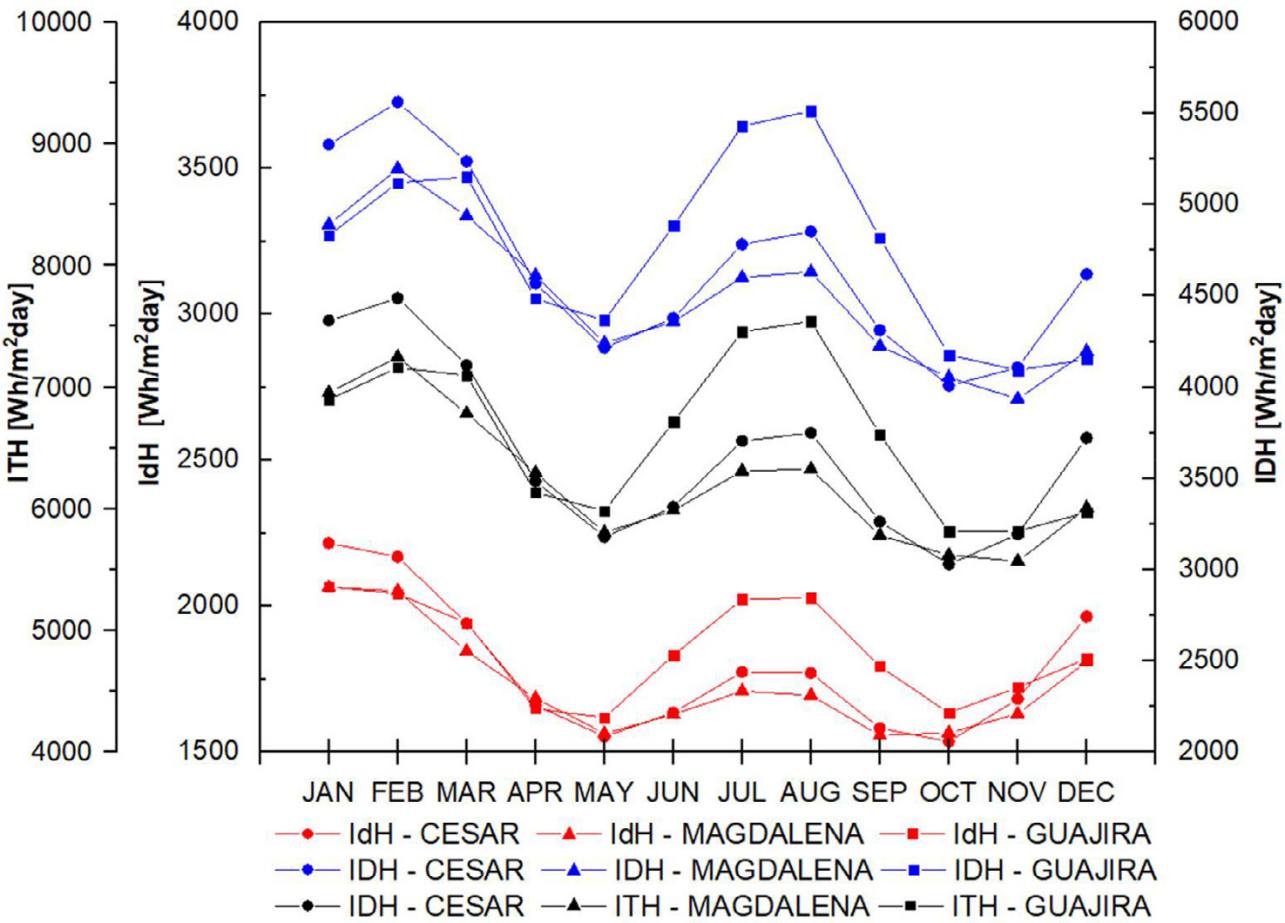
Analysis of direct, diffuse and total radiation at La Guajira, Magdalena and Cesar weather stations from 1993 to 2013 for a *τ*(0.2).

**Fig. 8:**
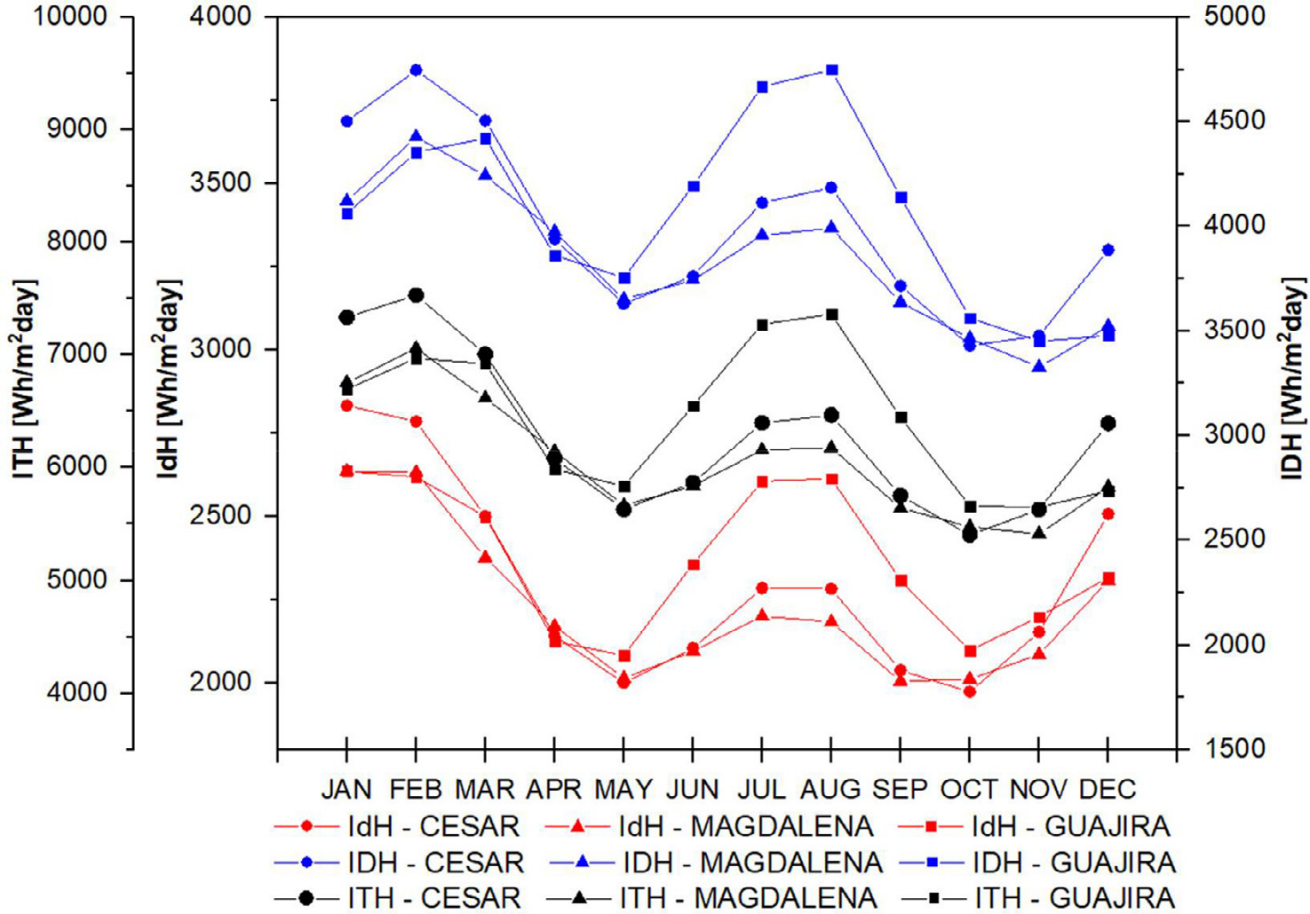
Analysis of direct, diffuse and total radiation at La Guajira, Magdalena and Cesar weather stations from 1993 to 2013 for a *τ*(0, 3).

**Fig. 9:**
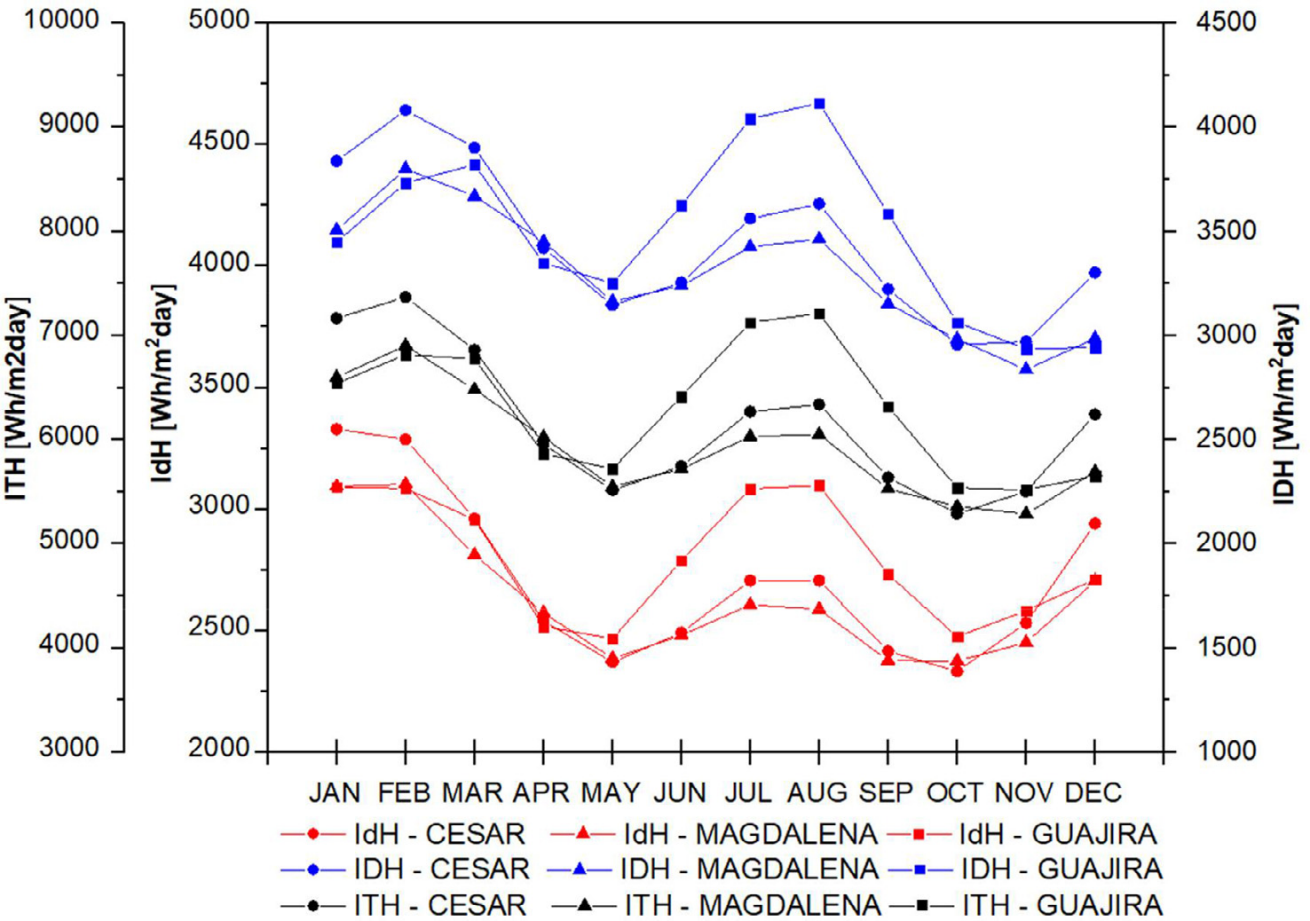
Analysis of direct, diffuse and total radiation at La Guajira, Magdalena and Cesar weather stations from 1993 to 2013 for a *τ*(0.4).

The data set is completed with the behavior of direct, diffuse and total solar radiation in the study departments considering the atmospheres presented in Fig. 5 for t(0.0), in Fig. 6 for t(0.1), in Fig. 7 for t(0.2), in Fig. 8 for t(0.3), and in Fig. 9 for t(0.4), based on data collected for 20 years every day. This collection process allowed us to determine the monthly behavior in every department considering all of their weather stations, as seen in the original data raw file in the attachment. The information of each column in the raw dataset is presented in Table 4. All raw data is available in https://data.mendeley.com/datasets/hytc559th5/1.

**Table 4:** Information of each column in the raw dataset.

Symbol	Description	Symbol	Description
Max	Maximun daily temperatura ( °C)	R7	Relative humidity at 7:00 a.m. (%)
Min	Minimun daily temperatura ( °C)	R13	Relative humidity at 1:00 p.m. (%)
Max-Min	Temperature difference, ( °C)	R19	Relative humidity at 7:00 p.m. (%)
T-7	Dry bulb temperature at 7:00 a.m. ( °C)	Mean	Mean daily relative humidity, (%)
T-13	Dry bulb temperature at 1:00 p.m. ( °C)	P7	Pressure at 7:00 a.m. (inHg)
T-19	Dry bulb temperature at 7:00 p.m. ( °C)	P13	Pressure at 1:00 p.m. (inHg)
Mean	Mean daily dry bulb temperature, ( °C)	P19	Pressure at 7:00 p.m. (inHg)
T7	Wet bulb temperature at 7:00 a.m. ( °C)	Mean	Mean daily pressure (inHg)
T13	Wet bulb temperature at 1:00 p.m. ( °C)	Tr7	Dew temperature at 7:00 a.m.
T19	Wet bulb temperature at 7:00 p.m. ( °C)	Tr13	Dew temperature at 1:00 p.m.
Mean	Mean daily wet bulb temperature, ( °C)	Tr19	Dew temperature at 7:00 p.m.

## 2 Experimental Design, Materials and Methods

### 2.1 Meteorological data

**Fig. 10:**
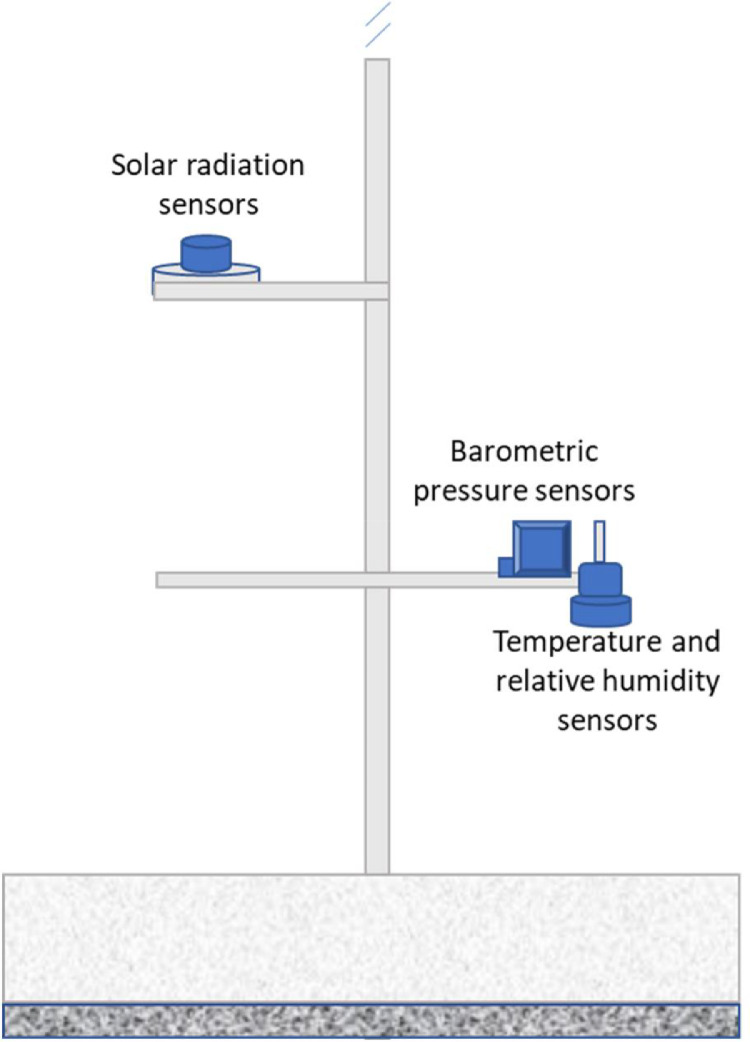
Weather station schematic diagram.

The meteorological stations are located in the departments of La Guajira, Magdalena, and Cesar. These departments are located at the coordinates 11 ° 14′31 ″ N 74 ° 12′19 ″ W, 11 ° 33′N 72 ° 54′W and 10 ° 29′00 ″ N 73 ° 15′00 ″ W. IDEAM provided the temperature, relative humidity, and pressure data required to carry out this study. These measurements were made through the sensors whose specifications are presented in Table 5 and Fig. 10.

**Table 5:** Sensors technical data.

Measurement	Precision
Barometric pressure	± 1hPa
Temperature	30 to +60 °C; ±0,3 °C
Relative humidity	±0,5% RH

### 2.2 Method

#### 2.2.1 Bird and Hulstrom model for calculating solar radiation

Taking into account the physical and meteorological conditions of the departments of Magdalena, La Guajira and Cesar, located on the Caribbean coast of Colombia, the Bird and Hulstrom model [2] were used, which is a physical and empirical model based on data and measurements taken in different stations over a certain time. From these measurements, it calculates the atmospheric transmittances that will allow the calculation of total, direct, and diffuse radiation. This model is considered the most suitable because it allows us to identify different coefficients responsible for radiation attenuation due to the presence of different particles in the atmosphere. This model determines direct (IDH) and diffuse (IdH) radiation to know in this way the value of total radiation (ITH).

The direct radiation on a horizontal surface is determined from Eq. (1), considering different cloudiness levels.

(1) $$I_{DH}=0.9662\cdot C_{r}\cdot \tau_{prom}\cdot sen\left(A\right)\;\left(W/m2\right)$$

where *τ_prom_* corresponds to the average transmittance calculated from the transmittance by air molecules dispersion (*τ_r_*), the transmittance by ozone molecules dispersion (*τ_o_*), the transmittance by miscible gasses molecules (*τ_g_*), the transmittance by water vapor (*τ_w_*), the transmittance by aerosol sprays molecules (*τ_a_*), it is also taken into account the daily solar constant which is a function of the Julian day measured in W/m^2^. Also, the 0.9662 belonging to the Eq. (1) is the correction factor adjusted to the wavelength Interval where 96% of the radiation is concentrated and (A) is the angle of solar altitude. For every one of these transmittance values, different parameters must be considered, which are related in Table 6.

**Table 6:** Parameters to be taken into account for the values of transmittances.

Transmission Coefficient	Parameters
Air molecules	Atmospheric mass (ma)
Miscible gasses	Atmospheric mass (ma)
Ozone	Thickness of the ozone layer
Water	Amount of precipitable water on site
Aerosols	Turbidity Coefficient (β)

The value (β) can vary from 0.0 for extremely clean atmospheres, until 0.4 as maximum limit for atmospheres with very high murkiness. Where there are no available measurements, like in this case, the expression proposed by Mächler [3] taken from Buckius and King can be used [4], represented by the Eq. (2), where VIS corresponds to the sky visibility value in km.

(2) $$\beta =0.55^{\alpha }\cdot \left(\;\frac{3.912}{\text{VIS}}-0.01162\right)\cdot \left[0.024722\cdot \left(\text{VIS}-5\right)+1.132\right]$$

Where α indicates the particle size, Mächler suggests as an approximate median value 1.3 μm, if it is about a natural atmosphere. According to Eq. (2), the values in the parameter β will give the visibility in km as shown in Table 7 [4].

**Table 7:** Sky visibility according to Angström coefficients (β).

β	0.0	0.1	0.2	0.3	0.4
km	340	30	11	7	<5

According to Global Learning and Observations to Benefit the Environment [5], it is defined that for β=0.0 the atmosphere is extremely clean in which the sky presents a deep blue color, unusual situation in Earth, so for this case of study a value of β=0.1 is taken, the atmosphere is clear, which indicates a cloudiness-free sky, of blue color, which has place only in determined occasions. For a β = 0.2, the atmosphere presents clear sky conditions with slight cloudiness, a characteristic that is more common and some authors identify them as a light blue sky, with some haze. For a β = 0.3 the atmosphere presents a degree of turbidity that indicates greater cloudiness, under these conditions it has a pale blue color, with more lime. For a β = 0.4, the atmosphere appears cloudy; in this case, the sky presents a "milky" color characteristic of extreme haze.

Regarding the diffuse radiation, the model considers three solar components, the *I_dr_* due to the existence of air molecules, *I_da_* due to the existence of dust particles, and *I_dm_* which is by multiple reflection between the soil and the atmosphere [6].

The diffuse radiation due to the existence of air molecules is described by the Eq. (3).

(3) $$I_{dr}=\left[\frac{0.79\cdot C_{r}\cdot \tau_{0}\cdot \tau_{g}\cdot \tau_{w}\cdot \tau_{\text{aa}}}{2}\right]\cdot \left[\frac{1-\tau_{r}}{1-m_{a}+m_{a}^{1.02}}\right]\cdot sen\left(A\right)\;\left(W/m2\right)$$

where *τ_aa_* corresponds the transmittance due to the absorption of aerosols which is a function of the air mass (ma) and the transmittance due to aerosols *τ_a_* used for the calculation of direct radiation.

In this model, the transmittance value by scattering (*τ_r_*) evaluates the change of direction that the solar radiation experiences due to the air molecules presence and it's determined from the Eq. (4).

(4) $$\tau_{r}=\;e^{-0.0903\cdot m_{a}^{0.84}\left(1+m_{a}\;{-\;m}_{a}^{1.01}\right)}$$

For their calculation, it is necessary to determine the optical mass of the air (*m_a_*) which is corrected by pressure as shown by the Eq. (5).

(5) $$,m_{a,}=\frac{,P_{,T}\cdot ,m_{,rel,}}{1,013,25}$$

Where *P_T_* es the total pressure of the air in Pa and it is determined in function of the altitude (z) with the Eq. (6) [6].

(6) $$P_{T}=101,325\cdot e^{-0.0001184\cdot z}$$

To calculate the air mass value, it is required to evaluate first the relative air mass value (m_rel_). This is determined by Eq. (7).

(7) $$m_{\text{rel}}=\;\frac{1}{\cos U+0.15\cdot {\left[93.885\;\cdot U\right]}^{-1.253}}\;$$

Diffuse radiation due to the presence of aerosol sprays, represented by the Eq. (8), is calculated from the C Model of Iqbal [7], which is function of the energy percentage that approaches to the Earth surface due to the aerosol spray dispersion (*F_c_*). In this case, its value can be estimated from the parametrization realized by Mac, whose calculation is function of the atmospheric mass (m_a_) [8].

(8) $$I_{da}=0.79\cdot C_{r}\cdot \tau_{0}\cdot \tau_{g}\cdot \tau_{w}\cdot \tau_{\text{aa}}\cdot F_{c}\cdot \left[\frac{1-\tau_{\text{as}}}{1-m_{a}+m_{a}^{1.02}}\right]\cdot sen\left(A\right)\;\left(W/m2\right)$$

In this equation the transmittance is used (*τ_as_*) which is due to the aerosol spray diffusion which is function of (*τ_a_*) and (*τ_aa_*).

The calculation of the diffuse radiation by multiple reflection, represented by the Eq. (9), requires having the surface reflection coefficients (*ρg*), this value is generally tabulated. In the same way, it is required to evaluate the atmospheric albedo; that is, the multiple reflection between the soil and the sky (*ρ’a*) which is function of *F_c_* and the transmittance due in exclusiveness to diffusion by aerosol sprays [9].

(9) $$I_{dm}=\left[I_{DH}\cdot sen\left(A\right)+I_{dr}+I_{da}\right]\cdot \left[\frac{\rho_{g}\cdot \rho_{a}}{1-\rho_{g}\cdot \rho_{a}}\right]\;W/m2)$$

As it was mentioned earlier, this model indicates that the solar irradiation is equivalent to the sum of the direct and diffuse irradiation as presented in the Eq. (10).

(10) $$I_{TH}\;=\;I_{DH}\;+\;I_{dH}$$

## CRediT authorship contribution statement

**Marley Vanegas Chamorro:** Conceptualization, Methodology, Validation, Writing - original draft. **Edwin Espinel Blanco:** Data curation, Funding acquisition. **Jhan Piero Rojas:** Formal analysis, Resources.

## Declaration of Competing Interest

The authors declare that they have no known competing financial interests or personal relationships that could have appeared to influence the work reported in this paper.

## Appendix Supplementary materials

Image, application 1

Image, application 2

Image, application 3

Image, application 4

Image, application 5

Image, application 6

Image, application 7

Image, application 8

Image, application 9

Image, application 10

Image, application 11

Image, application 12

Image, application 13

Image, application 14

Image, application 15

Image, application 16

Image, application 17

Image, application 18

Image, application 19

Image, application 20

Image, application 21

Image, application 22

Image, application 23

Image, application 24

Image, application 25

Image, application 26

Image, application 27

Image, application 28

Image, application 29

Image, application 30
